# Increase of Zinc Finger Protein 179 in Response to CCAAT/Enhancer Binding Protein Delta Conferring an Antiapoptotic Effect in Astrocytes of Alzheimer’s Disease

**DOI:** 10.1007/s12035-014-8714-9

**Published:** 2014-05-01

**Authors:** Shao-Ming Wang, Yi-Chao Lee, Chiung-Yuan Ko, Ming-Derg Lai, Ding-Yen Lin, Ping-Chieh Pao, Jhih-Ying Chi, Yu-Wei Hsiao, Tsung-Lin Liu, Ju-Ming Wang

**Affiliations:** 1 Institute of Basic Medical Sciences, College of Medicine, National Cheng Kung University, Tainan, 701 Taiwan; 2 Institute of Bioinformatics and Biosignal Transduction, College of Bioscience and Biotechnology, National Cheng Kung University, Tainan, 701 Taiwan; 3 Infectious Disease and Signaling Research Center, National Cheng Kung University, Tainan, 701 Taiwan; 4 Center of Molecular Inflammation, National Cheng Kung University, Tainan, 701 Taiwan; 5 Graduate Institute of Medical Sciences, Taipei Medical University, Taipei, 110 Taiwan; 6 Ph.D. Program for Neural Regenerative Medicine, College of Medical Science and Technology, Taipei Medical University, Taipei, 110 Taiwan; 7 Center for Neurotrauma and Neuroregeneration, Taipei Medical University, Taipei, 110 Taiwan

**Keywords:** Alzheimer’s disease, CEBPD, ZNF179, Astrocytes, Antiapoptosis

## Abstract

Reactive astrogliosis is a cellular manifestation of neuroinflammation and occurs in response to all forms and severities of the central nervous system (CNS)’s injury and disease. Both astroglial proliferation and antiapoptotic processes are aspects of astrogliosis. However, the underlying mechanism of this response remains poorly understood. In addition, little is known about why activated astrocytes are more resistant to stress and inflammation. CCAAT/enhancer binding protein delta (CEBPD) is a transcription factor found in activated astrocytes that surround β-amyloid plaques. In this study, we found that astrocytes activation was attenuated in the cortex and hippocampus of APPswe/PS1 E9 (*App*Tg)/*Cebpd* ^−/−^mice. Furthermore, an increase in apoptotic astrocytes was observed in *App*Tg/*Cebpd* ^−/−^mice, suggesting that CEBPD plays a functional role in enhancing the antiapoptotic ability of astrocytes. We found that Zinc Finger Protein 179 (ZNF179) was a CEBPD-regulated gene that played an antiapoptotic, but not proliferative, role in astrocytes. The transcriptions of the proapoptotic genes, insulin-like growth factor binding protein 3 (IGFBP3) and BCL2-interacting killer (BIK), were suppressed by ZNF179 via its interaction with the promyelocytic leukemia zinc finger (PLZF) protein in astrocytes. This study provides the first evidence that ZNF179, PLZF, IGFBP3, and BIK contributed to the novel CEBPD-induced antiapoptotic feature of astrocytes.

**Electronic supplementary material:**

The online version of this article (doi:10.1007/s12035-014-8714-9) contains supplementary material, which is available to authorized users.

## Introduction

Aging is accompanied by a low-grade chronic neuroinflammation and aged rat neurons show signs of programmed cell death [1, 2]. Astrocyte is the most abundant cell type of glial cells and suggests being involved in the induction of neuroinflammation. Previous studies reported no change in the number of astrocytes in the hippocampus of male aged mice [2] and activated astrocytes are more resistant to death signals in highly inflammatory environments [3, 4]. Interestingly, the number of astrocytes increases by approximately 20 % in aging brains [5], suggesting that reactive gliosis responds to injured or damaged neurons during aging. The above discoveries indicated that the antiapoptosis of aged astrocytes contributes to the death of neurons. However, the underlying mechanisms have not been investigated. Alzheimer’s disease (AD) is pathologically characterized by the age-dependent deposition of β-amyloid (Aβ) in senile plaques and is associated with neuroinflammation. Aβ can activate astrocytes, thus promoting inflammatory cytokines production, which is believed to modulate the development and/or progression of AD. Astrogliosis (also known as astrocytosis or reactive astrocytes) is characterized by the proliferation and hypertrophy of astrocytes, and is usually observed in neurodegenerative disorders and central nervous system (CNS) injuries [6].

CCAAT/enhancer binding protein delta (CEBPD) is a member of the CCAAT/enhancer binding protein (C/EBP) family and has been shown to be activated in many inflammatory diseases, including AD [7, 8] and rheumatoid arthritis [9]. CEBPD is responsive to tumor necrosis factor alpha (TNFα), interleukin-1 beta (IL-1β), interleukin 6 (IL-6), lipopolysaccharide, and interferon gamma [8, 10]. In astrocytes, CEBPD activation attenuates macrophage-mediated phagocytosis of damaged neurons and promotes chemoattraction and migration of microglia/macrophages through PTX3 and MCP-1, respectively [8, 11]. However, the function of CEBPD in astrocytes and neuroinflammation-related diseases, especially its potential role in astrogliosis, remains largely uninvestigated.

Zinc finger protein 179 (ZNF179 or RNF112) belongs to the RING finger protein family, which is characterized by a zinc binding domain that serves as a potent protein binding interface. Mouse ZNF179 (Znf179) is specifically expressed in the mouse brain [12, 13]. Znf179 is expressed exclusively in the mouse brain and the expression is high in MAP2-positive cells and moderate in glial fibrillary acidic protein (GFAP)-positive cells in normal subjects [14]. However, the ZNF179 biology in brain remains largely unclear. A recent study showed that Znf179 regulates cell cycle exit, which is critical for neuronal differentiation and plays a potent role in cell survival [12].

Herein, we found that the loss of GFAP signal associated with the increase of caspase 3 signal was observed in *App*Tg/*Cebpd* ^−/−^ mice. Focusing on the survival (or resistance to stress-induced apoptosis) of astrocytes in inflammatory environment, we first showed that CEBPD played a functional role in inducing the resistance of cell death in an inflammatory environment. ZNF179 is attenuated following the IL-1β stimulation in neuronal cells. We further found that ZNF179 is responsive to CEBPD induction in astrocytes and contributes the CEBPD-induced antiapoptosis. Two pro-apoptotic genes insulin-like growth factor binding protein 3 (IGFBP3) and BCL2-interacting killer (BIK) were identified by a system approach and were negatively regulated by the complex of ZNF179 and promyelocytic leukemia zinc finger (PLZF). These discoveries provide a new insight into the resistance of astrocytes to inflammation and the control of astrogliosis.

## Materials and Methods

### Materials

The CEBPD and green fluorescent protein (GFP) antibodies were purchased from Santa Cruz Biotechnology (Santa Cruz, CA, USA). The GFAP antibody was purchased from Invitrogen (Carlsbad, CA, USA). The caspase 3 antibody was purchased from Cell Signaling Technology (Danvers, MA, USA). The ZNF179 antibody used for immunofluorescence was purchased from GeneTex (Irvine, CA, USA), and the ZNF179 antibody used in the Western blot and immunoprecipitation assays was obtained from Dr. Yi-Chao Lee. The TRIzol RNA extraction reagent, Dulbecco’s modified Eagle’s medium (DMEM), and SuperScript™ III were purchased from Invitrogen (Carlsbad, CA, USA). All oligonucleotides were synthesized by MDBio Inc. (Taipei, Taiwan). Fetal bovine serum (FBS) was purchased from HyClone Laboratories (Logan, UT, USA).

### Animals

The APPswe/PS1/E9 bigenic (*App*Tg) mice were obtained from the Jackson Laboratory (stock no. 004462, Bar Harbor, ME, USA). The *App*Tg mice were crossed with *Cebpd*-deficient mice (*Cebpd* ^−/−^), a kind gift from Dr. E. Sterneck [15], on the C57BL/6 genetic background. Female mice heterozygous for *App*Tg mice was intercrossed with *Cebpd* ^−/−^ homozygous mice; the offspring (*App*Tg^+/−^/*Cebpd* ^+/−^) were then bred to each other to produce the *App*Tg/*Cebpd* ^−/−^ mice in this study.

### Cell Culture and Isolation of Primary Mouse Astrocytes

Human U373MG cells, an established cell line derived from human astrocytoma and HeLa cells (human cervical epithelioid carcinoma cell line), were cultured in DMEM. SH-SY5Y cells (human neuroblastoma cell line) were maintained in DMEM/F12. All media contained 10 % FBS, 100 μg/mL streptomycin, and 100 units/ml penicillin. U373MG cell lines stably expressing hemagglutinin (HA), HA-ZNF179, or HA-CEBPD were selected and maintained by regular media containing G418. The primary mouse brain astrocytes were isolated from *Cebpd* ^+/+^ or *Cebpd* ^−/−^ mice using mechanical dissociation of the brain cortex from newborn pups. The isolated cells were then filtered through a 70-μm nylon strainer and cultured in the previously described medium [16] with the addition of poly-l-lysine (Invitrogen, Carlsbad, CA, USA).

### Cell Survival, Proliferation, and Fluorescence-Activated Cell Sorting Analysis

For the cell survival assay, cells were plated and cultured in the aforementioned regular medium for 16 h. The experimental cells were then treated with or without 100 μg/mL methanesulfonate (MMS; Sigma, St. Louis, MO, USA) or 5 ng/mL IL-1β (Invitrogen, Carlsbad, CA, USA). Next, the media was removed and replaced with diluted 3-(4,5-cimethylthiazol-2-yl)-2,5-diphenyl tetrazolium bromide (MTT) reagent for 4 h. The samples were then measured spectrophotometrically at 595 nm using an ELISA plate reader. For cell proliferation assay, daily cell culture samples were counted in a Neubauer chamber after trypsinization. Viability was assessed by Trypan blue exclusion. Assays were performed in triplicates. For the apoptosis assay, cells were plated and cultured in the aforementioned regular medium for 16 h. The experimental cells were then treated with or without MMS or IL-1β for 24 h. As indicated times or experimental conditions, the cells were fixed in cold 70 % ethanol at −20 °C overnight and then suspended in phosphate-buffered saline (PBS) containing 0.2 % Triton X-100, 0.1 mg/ml RNase and 40 μg/mL propidium iodide at room temperature for 1 h. The apoptosis of experimental cells was analyzed using flow cytometry (FACSCalibur; BD Biosciences, Mountain View, CA).

### Reverse Transcription-PCR and Quantitative PCR

Total RNA was extracted using the TRIsure RNA extraction reagent. The synthesis of complementary DNA (cDNA) was completed with an reverse transcription (RT) reaction using SuperScript III. Quantitative PCR (Q-PCR) was conducted using KAPA SYBR FAST qPCR Master Mix (Life Technologies Corporation and Kapa Biosystems Inc.). PCR was conducted using a CFX connect real-time PCR system (Bio-Rad) with the following pairs of specific primers: primer sequences human CEBPD (S): 5-′GCCATGTACGACGACGAGAG-3′ and CEBPD (AS): 5-′TGTGATTGCTGTTGAAGAGGTC-3′; mouse Cebpd (S): 5-′CTCCCGCACACAACATACTG -3′ and Cebpd (AS): 5-′AGTCATGCTTTCCCGTGTTC-3′, ZNF179 (S): 5-′GAGCAGGGAAACAAGGATCA-3′ and ZNF179 (AS): 5-′GGTGGGATGAGTCACGATCA-3′, RYBP (S): 5-′TGACATTGCAGTGGTGGTTT-3′ and RYBP (AS): 5-′CCATGTCAGGACTGGATGTG-3′, BIK (S): 5-′CCTGGACCCTATGGAGGACT-3′ and BIK (AS): 5-′GGTGAAACCGTCCATGAAAC-3′, GADD45B (S): 5-′ACCTGCATTGTCTCCTGGTC-3′ and GADD45B (AS): 5-′TTTGTTTGTGGCAGCAACTC-3′, IGFBP3 (S): 5-′AGGGCACTCTGGGAACCTAT-3′ and IGFBP3 (AS): 5-′TGCAGTCATCCGAAGAATTG′.

### Western Blot Analysis

Cells were harvested and lysed with modified radioimmunoprecipitation assay (RIPA) buffer [50 mM Tris–HCl (pH 7.4), 150 mM sodium chloride, 1 mM ethylenediamine tetraacetic acid, 1 % NP40, 0.25 % sodium deoxycholate, 1 mM dithiothreitol, 10 mM NaF, 1 mM PMSF, 1 μg/mL aprotinin, and 1 μg/mL leupeptin]. Lysates were resolved on a sodium dodecyl sulfate-containing 10 % polyacrylamide gel, then transferred to a polyvinylidene difluoride nylon membrane and probed with primary antibodies for target proteins at 4 °C overnight. The specific proteins were detected by peroxidase-conjugated secondary antibody incubated at room temperature for 1 h. The signals were revealed by an enhanced chemiluminescence Western blot system from Pierce (Rockford, IL, USA).

### Luciferase Reporter Assay

The 5′ flanking regions of ZNF179, IGFBP3 and BIK genes were obtained by PCR with U373MG genomic DNA and then individually cloned into a pGL3 basic vector. The primers for the PCR of the genomic DNA were: ZNF179 (S): 5′-KpnI-GGGGTACCCCGCGCCAAGCCTATCACATATCC-3′, ZNF179 (AS): 5′-HindIII-CCCAAGCTTGGGCTGCGGTAGGTAGAAGGTGAGG-3′; IGFBP3(S): 5′-MluI-CGACGCGTCGAGTGCTACACTAACCAGTGGTC-3′, IGFBP3 (AS): 5′-HindIII-CCCAAGCTTGGGAATCCAGGCAGGAAGCGGCTGATC-3′, BIK(S):5′-NheI-CTAGCTAGCTAGCTCTTCCTCCTTTTGATCAGC-3′, and BIK (AS):5′-BglII-GAAGATCTTCTAAAACCTGGGCACGGCTC-3′. For the reporter assay, cells were transfected with the reporters and expression vectors as indicted using polyJet (SignaGen, Ijamsville, MD). The lysates of transfected cells were harvested following the manufacturer’s instructions for the luciferase assay.

### Co-Immunoprecipitation Assay

The lysates of U373MG cells were prepared using an immunoprecipitation lysis buffer [50 mM NaCl, 0.5 % NP-40, 10 mM Tris–HCl, (pH 8.0)]. The supernatant was collected and incubated with anti-GFP antibody at 4 °C for at least 4 h. Protein-A/G agarose beads were added to the lysates and the mixtures were incubated and rotated at 4 °C for 1 h. The beads were collected using centrifugation and washed three times with modified RIPA buffer. The proteins bound to the beads were eluted by adding 2X electrophoresis sample buffer and then subjected to Western blot analysis.

### Lentiviral Short Hairpin RNA Knockdown

The virus was produced from Phoenix cells using a co-transfection of the various short hairpin RNA (shRNA) expression vectors in combination with pMD2.G and psPAX2 expression vectors. The expression vectors were obtained from the National RNAi Core Facility located at the Genomic Research Center of Institute of Molecular Biology, Academia Sinica, Taiwan. After determining the viral infection efficiency, the lentivirus containing shβ-galactosidase (shLacZ), shCEBPD, or shZNF179 were used to infect U373MG cells for 48 h. The shRNA sequences in the lentiviral expression vectors were shLacZ, 5′-CCGGTGTTCGCATTATCCGAACCATCTCGAGATGGTTCGGATAATGCGAACATTTTTG-3′, shCEBPD, 5′-CCGGGCCGACCTCTTCAACAGCAATCTCGAGATTGCTGTTGAAGAGGTCGGCTTTTT-3′ and shZNF179, 5′-CCGGCTTCATGGACTCCTACACGATCTCGAGATCGTGTAGGAGTCCATGAAGTTTTTG-3′.

### Chromatin Immunoprecipitation Assay

Briefly, U373MG cells were treated with 1 % formaldehyde for 15 min. The cross-linked chromatin was then prepared and sonicated to an average size of 500 bp. The DNA fragments were immunoprecipitated with specific antibodies recognizing CEBPD, ZNF179, PLZF, or control rabbit immunoglobulin G (IgG) at 4 °C for 12–16 h. After reversal of the cross-linking between proteins and genomic DNA, the precipitated DNA was amplified by PCR with primers related to the specific regions on the genomic loci of target genes. The primers included ZNF179-I forward, 5′-GGGCTCTGTACATAGTAGGTACTC-3′ and ZNF179-I reverse, 5′-GCCTCTACTGGGCCAGCTGAGGTC-3′ or ZNF179-II forward, 5′-CTTGGTGCACCCATCTTTGCATC-3′ and ZNF179-II reverse, 5′-AGGCATTGCTATGATCTGGGTGAG-3′. IGFBP3 forward, 5′-CACGTGAGAGTCTTCTTGCGTTGAG-3′ and IGFBP3 reverse, 5′-ATACAGCGCTCCGCATTCGTGTG-3′, or BIK forward, 5′-CCCCTGGCTTCGGGTATGGATCAC-3′ and BIK reverse, 5′-TATCGGGGGGATCGATCCATTGAC-3′.

### Immunofluorescence Analysis

The frozen male mouse brain sections (~15–16 months of age) were treated with protein blocker/antibody diluents (Bio SB, Santa Barbara, CA, USA) for 1 h. In the same buffer solution, the sections were incubated overnight with primary antibodies at 4 °C. These primary antibodies included cleaved caspase 3, GFAP, Aβ, Znf179, and CEBPD. Statistical analysis of immunofluorescent staining intensity in cortex or hippocampus of brain slices from *App*Tg or *App*Tg/Cebpd^−/−^ mice was done using TissueQuest 4.0 image software. For the staining of cell cultures, U373MG cells were post-fixed in 4 % paraformaldehyde in PBS for 20 min, followed by 70 % methanol in PBS at −20 °C for 10 min. The fixed U373MG cells were further incubated with primary antibodies against target proteins in 3 % BSA at 4 °C. Pretreated slides of the tissue sections or U373MG cells were washed with 0.2 % Triton X-100 in PBS and then incubated with Alexa 488- or 555-conjugated secondary antibodies for 1 h at room temperature then washed again with 0.2 % Triton X-100 in PBS. Next, the glass slides were counter-stained and mounted with ProLong Gold antifade reagent with 4′,6-diamidino-2-phenylindole for immunofluorescence microscopy.

### Statistical Analysis

All experiments were repeated at least three times, and data were analyzed for statistical significance by two-tailed unpaired Student’s *t* test using Prism 5 software. The data were expressed as means ± SEM. Differences were considered statistically significant when indicated by asterisks.

## Results

### Astrocyte Activation is Attenuated in the Area Surrounding β-Amyloid Plaques in *App*Tg/*Cebpd*^−/−^ Mice

**Fig. 1:**
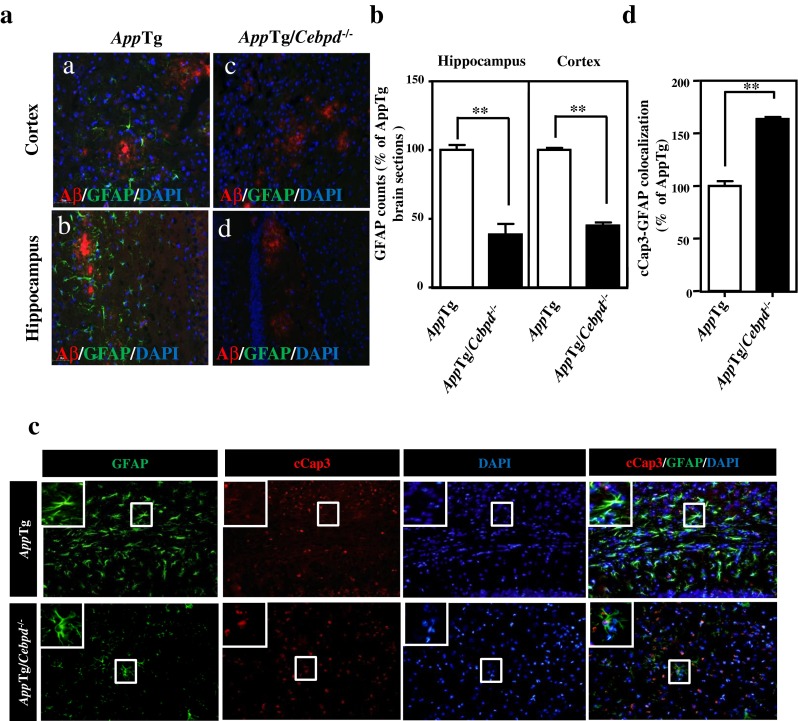
Astrocyte activation was decreased in *App*Tg/*Cebpd* ^−/−^ mice. **a** Astrocyte numbers were decreased in the area surrounding β-amyloid plaques in *App*Tg/*Cebpd* ^−/−^ mice. The brain tissue was subjected to immunofluorescence with anti-GFAP and anti-β-amyloid. **b** Quantitative analysis of GFAP staining in cortex and hippocampus brain sections of *App*Tg and *App*Tg/*Cebpd* ^−/−^ mice using TissueQuest software. **c** Loss of CEBPD promotes astrocyte death in *App*Tg/*Cebpd* ^−/−^ mice. The brain tissue was subjected to immunofluorescence with anti-GFAP and anti-cleaved caspase-3 (cCap3). **d** Quantitative analysis of GFAP and cCap3 immunofluorescent signal in whole brain sections of *App*Tg and *App*Tg/*Cebpd* ^−/−^ mice using TissueQuest software. (**p < 0.01, Student’s *t* test). *cCap3* cleaved caspase-3; *GFAP* glial fibrillary acidic protein

Activated astrocytes are largely resistant to cell death in a neuroinflammatory environment. In AD patients and *App*Tg mice, the expression of CEBPD and mouse CEBPD (Cebpd), respectively, is elevated and localized within astrocytes [7, 8] (Fig. S1A). The immunoreactivity for GFAP, a specific astrocyte marker, was attenuated in both the cortex and hippocampus of *App*Tg/*Cebpd* ^−/−^ mice when compared with *App*Tg mice (Fig. 1a, b). To assess whether apoptosis contributes to the reduction of astrocytes in *App*Tg/*Cebpd* ^−/−^ mice, activated caspase 3, an apoptosis marker, was used to measure the death of astrocytes in brain sections from *App*Tg and *App*Tg/*Cebpd* ^−/−^ mice. As shown in Fig. 1c, d, activated caspase 3 was upregulated and colocalized with the GFAP-positive astrocytes in *App*Tg/*Cebpd* ^−/−^ mice. This suggests that CEBPD contributes to the survival of astrocytes in *App*Tg mice.

### CEBPD Plays an Antiapoptotic Role in Astrocytes

**Fig. 2:**
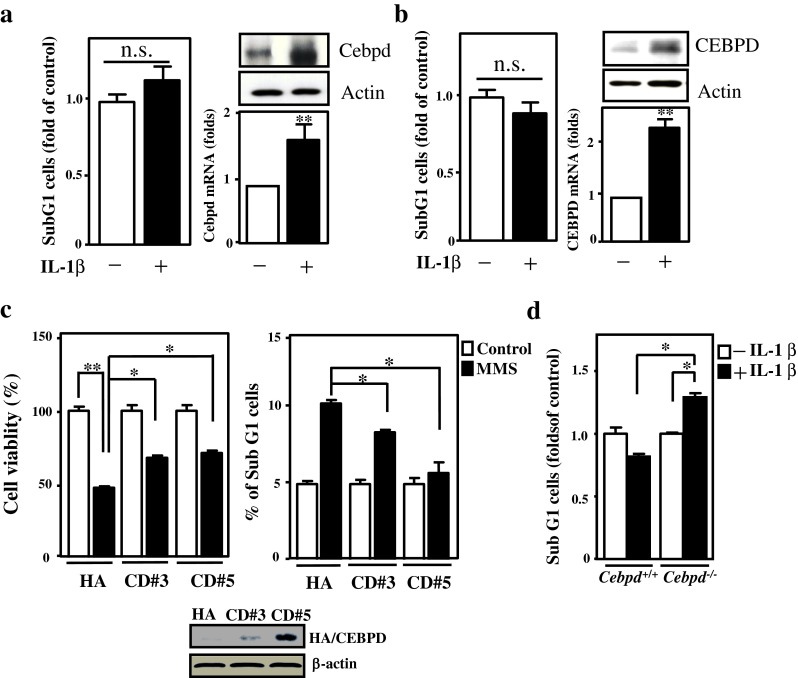
CEBPD promotes astrocyte survival and activation after exposure to inflammatory cytokines. **a** Primary mouse astrocyte and **b** U373MG cells are resistant to IL-1β-induced cell death. The dead cells were stained with PI and were localized to the sub-G1 population using flow cytometry. RT-PCR and Western blot analyses were conducted with specific primers and the indicated antibodies using total RNA and protein lysates harvested from IL-1β-treated cells. **c** CEBPD protects U373MG cells against MMS-induced death. The MTT assay and PI staining were conducted as indicated. **d** Loss of CEBPD in primary astrocyte cultures increased cell death after IL-1β treatment. The PI staining was conducted as indicated. (*p < 0.05, **p < 0.01, Student’s *t* test). *CD* CEBPD; *n.s*. not significant

Several reports have suggested that astrocytes are more resistant to death in an inflammatory environment when compared to neurons [3, 17, 18]. CEBPD is known to be activated in astrocytes in response to IL-1β and TNFα [8, 11]. We first tested whether the activated CEBPD contributes to the antiapoptosis of astrocytes. After 24 h of IL-1β treatment, CEBPD indeed responded to IL-1β stimulation and the level of apoptosis in U373MG and primary mouse astrocytes was not significantly different from that before treatment (Fig. 2a, b). In contrast to astrocytes, we found decreased CEBPD in and increased death of neuronal SH-SY5Y cells after 24 h of IL-1β treatment (Fig. S1B). To assess if CEBPD plays an antiapoptotic role in astrocytes, we generated U373MG cells that stably expressed CEBPD. As shown in Fig. 2c, the overexpression of CEBPD in U373MG cells increased survival and reduced apoptosis after exposure to MMS, a strong apoptosis inducer. Importantly, in *Cebpd* ^+/+^ and *Cebpd* ^−/−^ primary astrocytes, the cells lacking CEBPD showed increased cell death after IL-1β treatment (Fig. 2d). These data suggest that increased CEBPD in astrocytes has a functional role in antiapoptosis

### ZNF179 Gene is a Direct Target of CEBPD

**Fig. 3:**
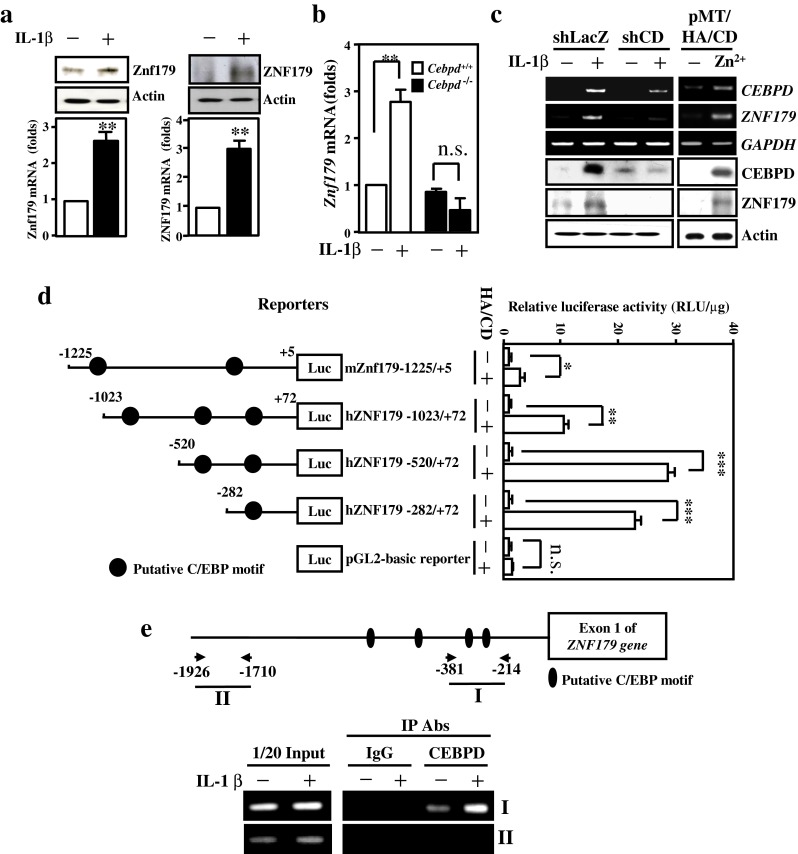
CEBPD directly regulates *ZNF179* transcription in U373MG cells. **a** Primary mouse astrocytes (*left panel*) and U373MG cells (*right panel*) were responsive to IL-1β treatment. RT-PCR and Western blot analyses were conducted with specific primers and the indicated antibodies using total RNA and protein lysates harvested from IL-1β-treated cells. **b** The lack of *Cebpd* did not induce Znf179 expression in primary astrocyte cultures. A Q-PCR assay was performed using total RNA harvested from *Cebpd* ^+/+^ and *Cebpd* ^−/−^ primary astrocytes. **c** CEBPD participates in IL-1β-induced *ZNF179* transcription. RT-PCR and Western blots were performed with total RNA and protein lysates harvested from stable U373MG cells with pMT-CEBPD expression vector (*right panel*) or IL-1β-treated U373MG cells with or without attenuation of CEBPD (*left panel*). **d** The identification of CEBPD responsive motifs on the *ZNF179* promoter region. The representation of reporter constructs (*upper panel*). A reporter assay was conducted using the luciferase activity of the *ZNF179* reporter/CEBPD expression vector co-transfected cell lysates. **e** CEBPD directly binds to the *ZNF179* promoter in vivo. A chromatin immunoprecipitation assay was performed with the immunoprecipitation products at the indicated Abs from U373MG cells treated with IL-1β. (*p < 0.05, **p < 0.01, ***p < 0.001, Student’s *t* test). *shCD* short hairpin CEBPD; *HA*/*CD* HA-tagged CEBPD; *n.s*. not significant; *IP* immunoprecipitation; *Abs* antibodies

We found that the expression of ZNF179 was responsive to IL-1β treatment in astrocytes (Fig. 3a). Furthermore, the expression of ZNF179 paralleled that of CEBPD in astrocytes (Fig. S1C). We next sought to determine the relationship between ZNF179 and CEBPD. A reporter assay showed that Cebpd could activate a *Znf179* promoter-driven reporter, but the expressed Znf179 had no effect on a *Cebpd* promoter-driven reporter (Fig. S2). These data suggest that CEBPD is an upstream regulator of the ZNF179 gene in astrocytes. Moreover, the effect of IL-1β-induced *Znf179* transcription was attenuated in primary astrocyte cultures derived from *Cebpd* ^−/−^ mice (Fig. 3b). A similar result was found for CEBPD-related *ZNF179* transcription and expression in U373MG cells (Fig. 3c). Using a serial deletion reporter assay, we identified a potent CEBPD responsive region in the *ZNF179* promoter at −282/+72 bp (Fig. 3d). In addition, an in vivo DNA binding assay showed that the binding of CEBPD on the *ZNF179* promoter was responsive to IL-1β in U373MG cells (Fig. 3e). These data suggest that CEBPD regulates *ZNF179* transcription by directly binding to the *ZNF179* promoter region.

### ZNF179 Expression in Astrocytes Contributes to Antiapoptosis

**Fig. 4:**
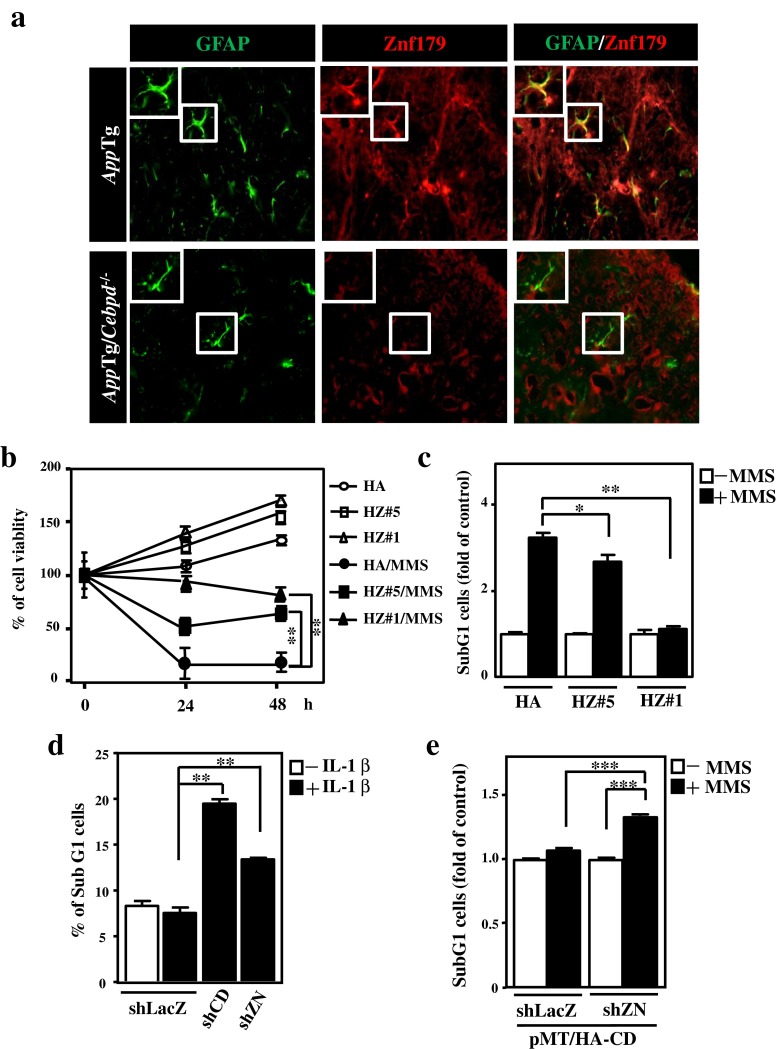
ZNF179 attenuates the IL-1β/MMS-induced death of U373MG cells. **a** Znf179 is highly expressed in the astrocytes of *App*Tg mice when compared with *App*Tg/*Cebpd* ^−/−^ mice. Sagittal sections of brain cortex were prepared from *App*Tg and *App*Tg/*Cebpd* ^−/−^ mice and then subjected to immunofluorescence with anti-GFAP, and anti-Znf179 antibodies. **b** and **c** ZNF179 protects U373MG cells against MMS-induced death. The MTT assay and PI staining were conducted as indicated. **d** Loss of CEBPD or ZNF179 increased cell death after IL-1β treatment. The PI staining was conducted as indicated. **e** The knockdown of ZNF179 in CEBPD overexpressing cells increased cell death after MMS treatment. The PI staining was conducted as indicated (*p < 0.05, **p < 0.01, ***p < 0.001, Student’s *t* test). *HZ* HA-tagged ZNF179; *shCD* short hairpin CEBPD; *shZN* short hairpin ZNF179; *GFAP* glial fibrillary acidic protein; *HA*/*CD* HA-tagged CEBPD; shLacZ, shβ-galactosidase

Although a previous study suggested that ZNF179 might contribute to the survival of neurons [12], the effect of ZNF179 in astrocytes is unknown. ZNF179 has been suggested to be a brain-specific gene [12, 19]. An immunofluorescence assay showed that ZNF179 was detectable in the GFAP-positive astrocytes of *App*Tg mice and was attenuated in the astrocytes of *App*Tg/*Cebpd* ^−/−^ mice (Fig. 4a). To determine if ZNF179 plays an antiapoptotic role, we established the stable expression of ZNF179 in human cervical HeLa cells. After MMS or TNFα exposure, HeLa cells with ZNF179 showed a resistance to MMS- or TNFα-induced apoptosis (Fig. S3A and S3B). A similar result was observed in U373MG cells stably expressing ZNF179 (Fig. 4b, c). A gain-of-function approach was used to assess if the lack of ZNF179 or CEBPD impacts the IL-1β-induced apoptosis of astrocytes. U373MG cells with reduced ZNF179 showed a loss of CEBPD and increased apoptosis after IL-1β exposure (Fig. 4d). Furthermore, the attenuation of ZNF179 decreased the CEBPD-related antiapoptotic effect after MMS exposure (Fig. 4e). These results suggest that ZNF179 is expressed in the astrocytes of *App*Tg mice, and it contributes to astrocytic CEBPD-dependent antiapoptotic effects.

### The Identification of ZNF179-Regulated Genes in U373MG Cells

**Fig. 5:**
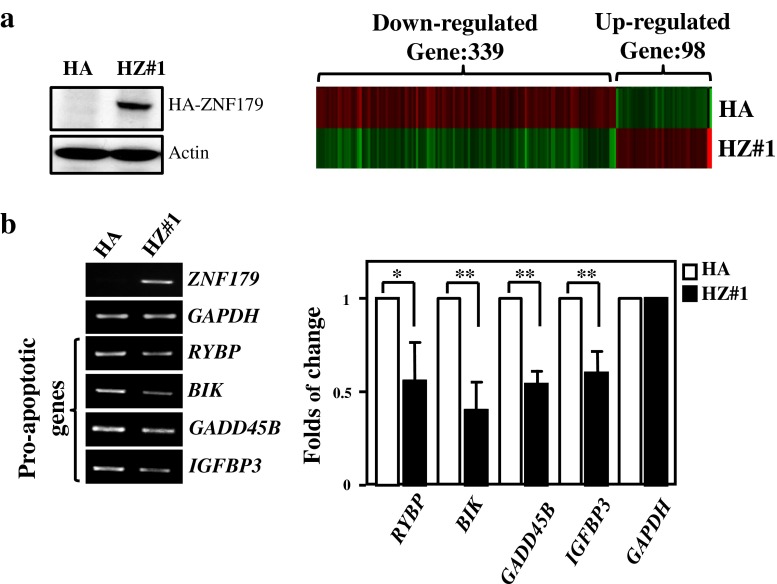
The identification of ZNF179-regulated genes in astrocytes. **a** A microarray analysis was performed using total RNA harvested from stable U373MG cells with a pcDNA-HA-ZNF179 expression vector. **b** The verification of CEBPD responsive genes in antiapoptosis. RT-PCR and Q-PCR assays were conducted using total RNA harvested from HA and HZ U373MG cells. (*p < 0.05, **p < 0.01, Student’s *t* test). *HZ* HA-tagged ZNF179

We demonstrated that ZNF179 plays a functional role in antiapoptosis. However, the ZNF179-responsive genes related to the antiapoptotic processes in astrocytes remain unknown. Genome-wide profiling and comparisons were conducted using U373MG cells with and without the stable expression of HA/ZNF179. As shown in Fig. 5a, we found that a total of 98 genes were significantly induced by ZNF179 and 339 genes were inhibited by ZNF179 in U373MG cells. Among these ZNF179-responsive genes, four proapoptotic genes, including *RYBP* [20], *BIK* [21], *GADD45B* [22] and *IGFBP3* [23, 24], were downregulated by ZNF179. RT-PCR and Q-PCR assays confirmed the findings from the microarray profile (Fig. 5b).

### ZNF179 Inhibits IGFBP3 and BIK Expression by Interacting with PLZF in Astrocytes

**Fig. 6:**
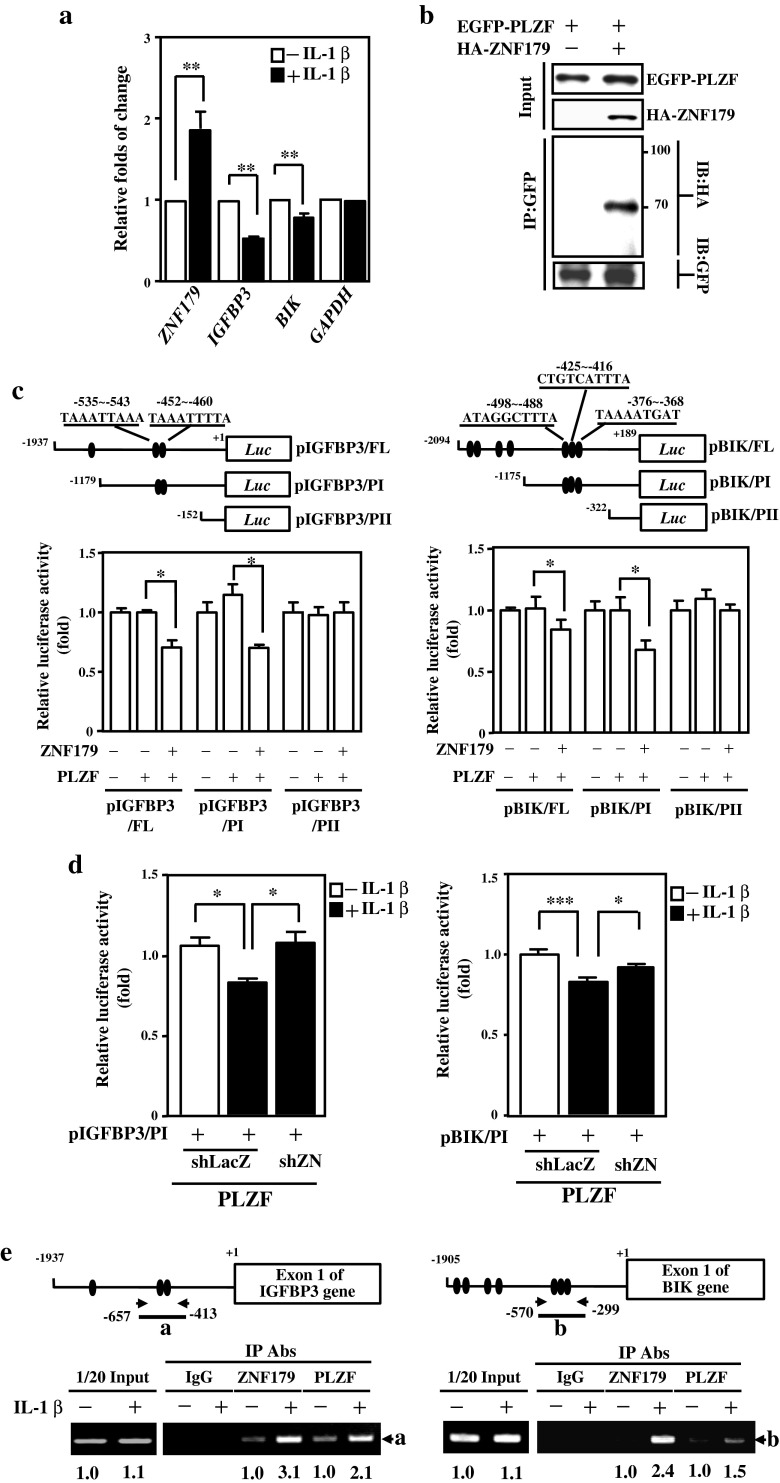
ZNF179 downregulates IGFBP3 and BIK expression through interaction with PLZF. **a** IGFBP3 and BIK expression was downregulated after IL-1β treatment. The Q-PCR assay was performed using total RNA harvested from U373MG cells. **b** The interaction of ZNF179 and PLZF was revealed using immunoprecipitation analysis of an EGFP-PLZF plasmid transiently transfected together with HA or HA-ZNF179 plasmids. **c** The interaction of ZNF179 with PLZF can downregulate *IGFBP3* and *BIK* promoter activity. A representation of reporter constructs are shown (*upper panel*). Using cell lysates, the reporter assay was conducted by examining the luciferase activity of a co-transfected *IGFBP3* reporter or *BIK* reporter/ZNF179 expression vector/PLZF expression vector. **d** ZNF179 reduces *IGFBP3* reporter or *BIK* reporter activities after IL-1β treatment in U373MG cells. Lentiviral vectors were used to express shLacZ and shZNF179. After a 2-day infection, these constructs were co-transfected with expression and promoter vectors. A reporter assay was conducted using the luciferase activity within cell lysates. **e** The interaction of ZNF179 with PLZF can directly binds to the *IGFBP3* and *BIK* promoter in vivo. A chromatin immunoprecipitation assay was performed with the immunoprecipitation products at the indicated Abs from U373MG cells treated with IL-1β. (*p < 0.05, **p < 0.01, ***p < 0.001, Student’s *t* test). *shZN* short hairpin ZNF179; *FL* full length; *PI* promoter I; *PII* promoter II; *shLacZ* shβ-galactosidase

We next sought to determine if these ZNF179-regulated genes were also responsive to IL-1β. By Q-PCR analysis, we found that only the *IGFBP3* and *BIK* transcripts out of the four ZNF179-responsive proapoptotic genes were downregulated in IL-1β-treated astrocytes (Fig. 6a). PLZF protein belongs to the family of Krüppel-like zinc finger proteins. Our recent study showed that ZNF179 can interact with PLZF in neurons [25]. Here, a Co-immunoprecipitation assay showed that ZNF179 also interacted with PLZF in astrocytes (Fig. 6b). Focusing on IGFBP3 and BIK, a serial deletion of *IGFBP3* and *BIK* promoters were cloned into a pGL3 basic vector. We identified two putative PLZF binding motifs at −535/−543 and −452/−460 of the *IGFBP3* promoter region and three motifs at −498/−488, −425/−416, and −376/−368 of the *BIK* promoter region (Fig. 6c, upper panel). Using a reporter assay, we found that overexpression of PLZF repressed the activities of *IGFBP3* and *BIK* reporters containing potent PLZF binding sites. The co-transfection of PLZF and ZNF179 expression vectors showed an extensive effect on the repression of *IGFBP3* and *BIK* reporter activity (Fig. 6c, bottom panel). Furthermore, the loss of ZNF179 reduced the PLZF-mediated repression of *IGFBP3* and *BIK* reporter activity after IL-1β exposure (Fig. 6d). To verify if the *IGFBP3* and *BIK* genes were directly regulated via the binding of PLZF and ZNF179, lysates of IL-1β-treated U373MG cells were subjected to a chromatin immunoprecipitation assay. Our results show that the binding of PLZF and ZNF179 to the *IGFBP3* and *BIK* promoter was responsive to IL-1β in U373MG cells (Fig. 6e). These data suggest that PLZF is an important component of IL-1β-induced ZNF179-mediated suppression of *IGFBP3* and *BIK* transcription.

## Discussion

In addition to AD, neuroinflammation has been suggested to play a critical role in the pathogenesis of several neurodegenerative disorders, including Parkinson’s disease and Huntington’s disease [26]. Activation of astrocytes and microglia has been associated with the pathogenesis of AD. However, the precise mechanisms involved in this process remain largely unclear. CEBPD is critical for the activation of astrocytes, but not for the microglia and neurons that surround Aβ plaques in AD (Fig. S1A) [7, 8, 11]. Under oxidative stress or inflammation, activated astrocytes were more resistant to apoptosis when compared with neurons (Fig. S1A and 2A) [3]. Our previous study showed that a significant reduction in astrocytes was observed in *App*Tg/*Cebpd* ^−/−^ mice [11]. In this study, we demonstrated that the loss of Cebpd promoted the death of GFAP-positive cells in the cortex and hippocampus of *App*Tg mice partly through the induction of apoptosis (Fig. 1c). Moreover, we demonstrated that the increase of CEBPD in astrocytes reduced MMS- or IL-1β-induced apoptosis (Fig. 2). These data suggest that CEBPD plays an antiapoptotic role in astrocytes, especially in a stress environment.

Elevated CEBPD expression has been mainly found in the astrocytes of AD patients and *App*Tg mice [7, 8, 11]. We determined that the activation of CEBPD in astrocytes results in attenuation of macrophage-mediated phagocytosis of damaged neurons, and also contributes to microglial activation and migration [8, 11]. In response to damaged or inflammatory environments, neurons are more sensitive than astrocytes because they have lower antioxidant capabilities and are therefore prone to excitotoxicity [27]. Our novel findings suggest that astrocytic CEBPD plays an antiapoptotic role, which contributes to the resistance of inflammation-induced apoptosis in the inflamed brain. Meanwhile, we also showed the first evidence to support ZNF179’s antiapoptotic role, particularly in astrocytes within a damaged or inflammatory environment.

CEBPD is induced in age-associated disorders, such as AD, atherosclerosis [28], type 2 diabetes [29], and rheumatoid arthritis [9, 30]. These discoveries imply that CEBPD may play a central role in inflammation-related diseases. Our previous [8, 11] and current studies indicate that the inactivation of CEBPD in astrocytes could improve the survival of neuron in inflammatory diseases. Small chemical drugs (<400 Da) may cross the blood–brain barrier via lipid-mediate-free diffusion [31]. Carnosic acid can penetrate the blood–brain barrier and could be a useful drug for Aβ-induced neurodegeneration as AD [32]. We previously demonstrated that rosmanol (molecular weight 346.41 Da), a natural carnosic acid, derived from the herb rosemary and inotilone (molecular weight 218.21 Da), an unusual 5-methyl-3(2H)-furanone derivative, can inhibit CEBPD activation in macrophages [9]. Therefore, rosmanol and inotilone can be tested toward the prevention or treatment of AD in the future.

Znf179 is specifically expressed in the brain and can contribute to neuronal differentiation and survival [12]. Unlike neurons, the increase of ZNF179 in astrocytes was responsive to inflammatory cytokines (Figs. 2, 3, and S1C). Here, we found that the transcription of *ZNF179* gene is responsive to IL-1β through the action of enhanced binding and transactivation activity of CEBPD on its promoter region in astrocytes. Importantly, the antiapoptotic role of ZNF179 in astrocytes may explain why active astrocytes are resistant to cell death in an inflammatory environment. Interestingly, the GSK3β inhibitor, LiCl, enhanced neuron survival but caused growth retardation in astrocytes [33]. A study showed that CEBPD is a GSK3β substrate, and CEBPD phosphorylation contributes to the transcriptional regulation of CEBPD-mediated downstream target genes. Therefore, the relevance of GSK3β-mediated phosphorylation of CEBPD in *ZNF179* transcriptional activation in astrocytes requires further investigation.

PLZF has been suggested to be a transcriptional repressor and antiapoptotic for neurons in stroke [34]. PLZF interacts with SMRT/N-CoR-mSin3A-HDAC-repressing complexes to suppress gene transcription [35, 36]. Several lines of evidence suggest that PLZF has antiapoptotic effects. For example, PLZF can inhibit caspase 3 and caspase 7 activity in neurons [34]; however, the specific mechanism remains unknown. In this study, we further found that ZNF179 expression was activated and interacted with PLZF upon IL-1β treatment to promote antiapoptosis in astrocytes. IGFBP3 is a common pro-apoptosis gene, and it has been shown to enhance and attenuate the actions of IGF-I [37]. IGF-I is mediated by the IGF-I receptor and interacts with IGFBP3 to inhibit cell growth and induce apoptosis [24]. Previous studies showed that IGFBP3 activates the apoptotic pathway through Bcl2 family [38]. Increased PLZF attenuated *IGFBP3* transcription. A constitutive binding of PLZF and increased binding of ZNF179 on the *IGFBP3* promoter were observed after IL-1β treatment. However, the nuclear-cytoplasmic shuttle of ZNF179 did not change in response to IL-1β stimulation (Fig. S4). This observation implies that cytosolic and nuclear ZNF179 may play distinct roles in IL-1β-treated astrocytes. These data also suggest that IL-1β can induce nuclear ZNF179 binding to the preexisting PLZF on *IGFBP3* and *BIK* promoter to suppress its transcription. Although the transcription of the proapoptotic *GADD45B* and *RYBP* genes is repressed by the overexpression of ZNF179, our preliminary results show that exposure to IL-1β has no effect on *GADD45B* and *RYBP* transcription. These data suggest that ZNF179 can interact with other IL-1β-independent transcription repressors or complexes to repress gene transcription; however, this hypothesis needs to be further investigated

## Electronic supplementary material

Below is the link to the electronic supplementary material.

ESM 1 (PPTX 21772 kb)
