# Supplementary material for: Increase of Zinc Finger Protein 179 in Response to CCAAT/Enhancer Binding Protein Delta Conferring an Antiapoptotic Effect in Astrocytes of Alzheimer’s Disease
Source: Mol Neurobiol. 2014 May 1;51(1):370–82. doi: 10.1007/s12035-014-8714-9 (PMC4309906; doi:10.1007/s12035-014-8714-9)
Supplement: Supplementary file 1 — (PPTX 21772 kb) [file 12035_2014_8714_MOESM1_ESM.pptx]

## Slide 1
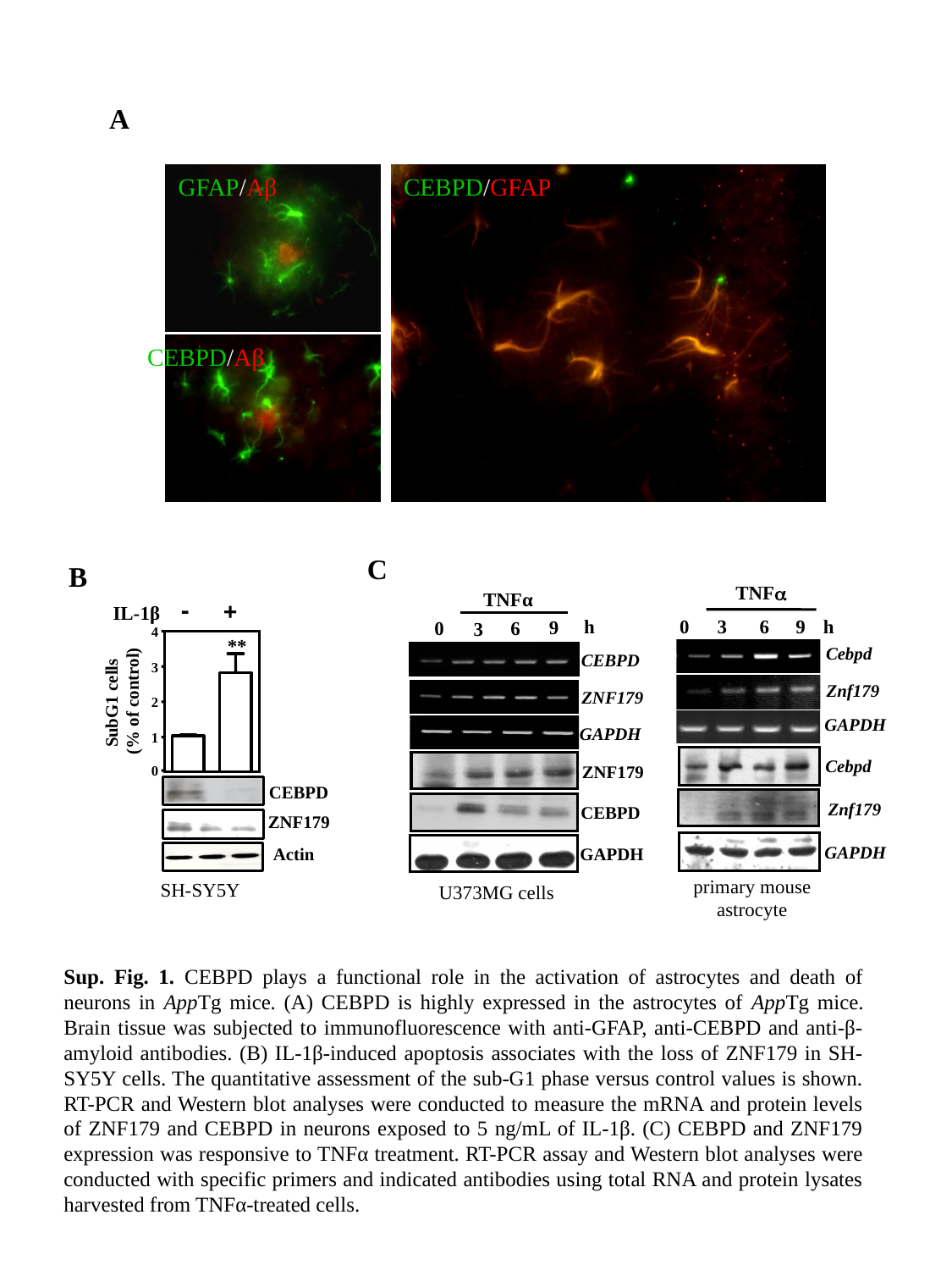

A
CEBPD/GFAP
GFAP/Aβ
CEBPD/Aβ
C
B
TNFa
TNFα
-
+
IL-1β
9
h
h
0
3
6
9
0
6
3
4
**
Cebpd
CEBPD
3
SubG1 cells
(% of control)
Znf179
ZNF179
2
GAPDH
GAPDH
1
Cebpd
ZNF179
0
CEBPD
Znf179
CEBPD
ZNF179
GAPDH
GAPDH
Actin
primary mouse astrocyte
SH-SY5Y
U373MG cells
Sup. Fig. 1. CEBPD plays a functional role in the activation of astrocytes and death of neurons in AppTg mice. (A) CEBPD is highly expressed in the astrocytes of AppTg mice. Brain tissue was subjected to immunofluorescence with anti-GFAP, anti-CEBPD and anti-β-amyloid antibodies. (B) IL-1β-induced apoptosis associates with the loss of ZNF179 in SH-SY5Y cells. The quantitative assessment of the sub-G1 phase versus control values is shown. RT-PCR and Western blot analyses were conducted to measure the mRNA and protein levels of ZNF179 and CEBPD in neurons exposed to 5 ng/mL of IL-1β. (C) CEBPD and ZNF179 expression was responsive to TNFα treatment. RT-PCR assay and Western blot analyses were conducted with specific primers and indicated antibodies using total RNA and protein lysates harvested from TNFα-treated cells.

## Slide 2
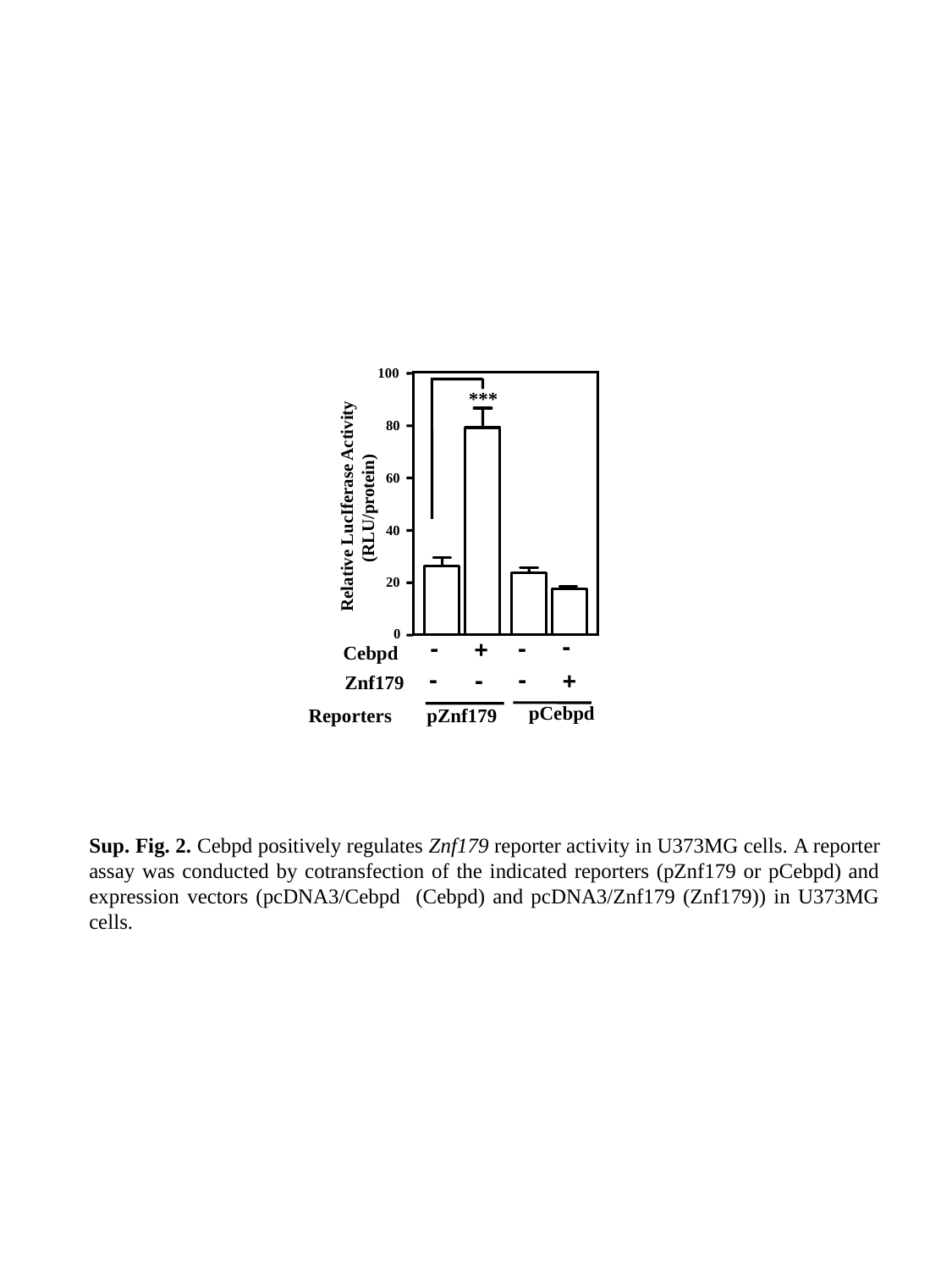

100
***
80
60
40
20
0
Relative LucIferase Activity
(RLU/protein)
-
-
-
+
Cebpd
-
-
-
+
Znf179
pCebpd
pZnf179
Reporters
Sup. Fig. 2. Cebpd positively regulates Znf179 reporter activity in U373MG cells. A reporter assay was conducted by cotransfection of the indicated reporters (pZnf179 or pCebpd) and expression vectors (pcDNA3/Cebpd (Cebpd) and pcDNA3/Znf179 (Znf179)) in U373MG cells.

## Slide 3
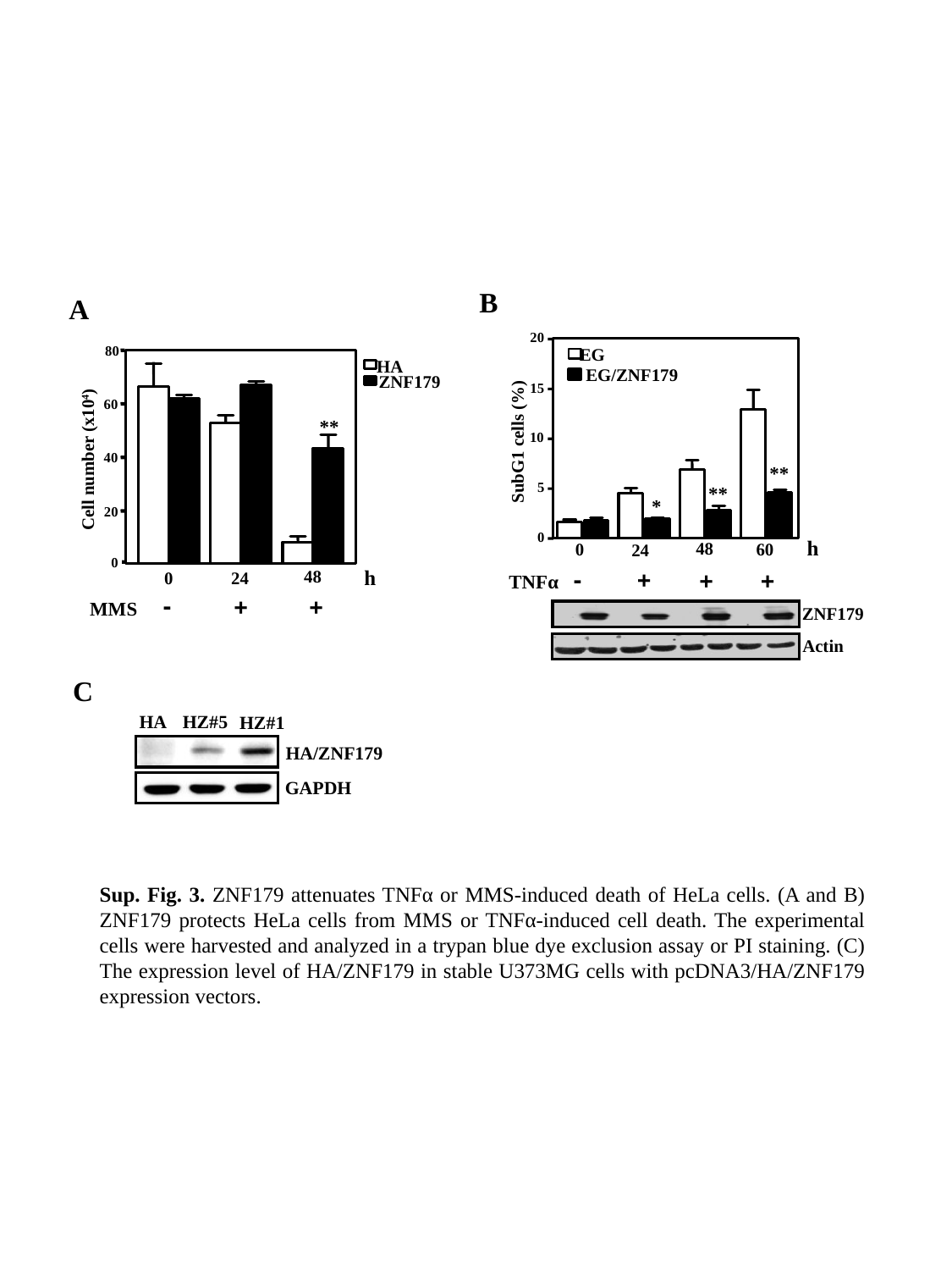

B
A
20
EG
EG/ZNF179
15
10
SubG1 cells (%)
5
0
h
48
60
0
24
-
+
+
+
TNFα
ZNF179
Actin
80
HA
ZNF179
60
Cell number (x104)
40
20
0
h
48
24
0
-
+
+
MMS
**
**
**
*
C
HA
HZ#5
HZ#1
HA/ZNF179
GAPDH
Sup. Fig. 3. ZNF179 attenuates TNFα or MMS-induced death of HeLa cells. (A and B) ZNF179 protects HeLa cells from MMS or TNFα-induced cell death. The experimental cells were harvested and analyzed in a trypan blue dye exclusion assay or PI staining. (C) The expression level of HA/ZNF179 in stable U373MG cells with pcDNA3/HA/ZNF179 expression vectors.

## Slide 4
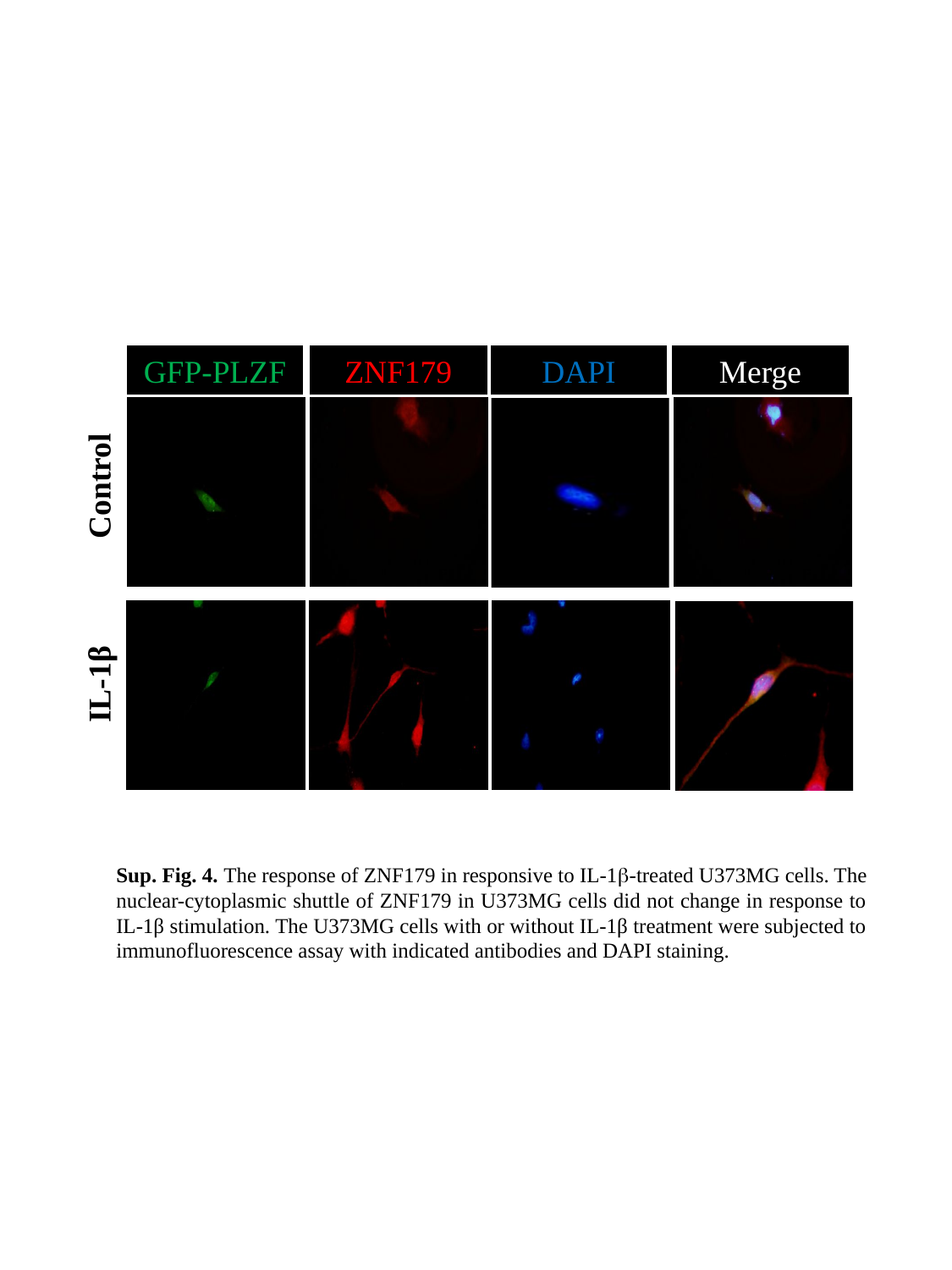

GFP-PLZF
ZNF179
DAPI
Merge
Control
IL-1β
Sup. Fig. 4. The response of ZNF179 in responsive to IL-1b-treated U373MG cells. The nuclear-cytoplasmic shuttle of ZNF179 in U373MG cells did not change in response to IL-1β stimulation. The U373MG cells with or without IL-1β treatment were subjected to immunofluorescence assay with indicated antibodies and DAPI staining.
